# Digitalis in Cardiac Dropsy

**Published:** 1875-04

**Authors:** 


Digitalis in Cardiac Dropsy.—Mr. I., aged 63, was suffering from dropsy, due to two abnormal conditions of his heart—mitral insufficiency and dilatation. He was treated with digitalis, about fifteen drops three times a day, morphia at night, etc. The dropsy was partially removed several times by the free use of elaterium, but quickly returned. He was now too debilitated to undergo a copious purgation. Moving his legs, even, was impossible. Pulse 130 and very feeble; respiration rapid and labored; at times a fierce, short struggle would ensue for more air; face cyanosed; recumbent position impossible. The dropsy, as stated, was general, and near its maximum; the skin of his legs presented a purplish discoloration, and was stretched to its utmost tension; cough persistent and very troublesome, due, no doubt, to the infiltration of water in the lung-tissue. The patient, in short, was drowning, and, unless speedily relieved, must die. Urine albuminous and scanty. Ordered one and one-half grains of digitalis with one grain of squill every two hours. In thirty-six hours the pulse was down to 70—a full, soft wave greeting my finger; urine passing in immense quantities. Patient felt much better, and was sorry he did not have a grist-mill to run, since he could furnish the water-power. Medicine reduced to six administrations a day, then four, three, two, and one. In eight days the dropsy had entirely disappeared, leaving the man almost a "living skeleton." His attenuated frame and wrinkled face told of the intense physical suffering endured before digitalis, in *doses large and oft repeated*, beat back the tide and manned the pumps. He sleeps well, eats well, and, with tonics and a liberal diet, is rapidly regaining his strength. There is now a buoyancy in the old man's step, and a warmth in his hearty grasp, contrasting strongly with the utter helplessness of his former state and the snaky coldness of his dropsied hands.—*Medical Times, January* 23.

